# Skin Lesions in Feline Leishmaniosis: A Systematic Review

**DOI:** 10.3390/pathogens10040472

**Published:** 2021-04-13

**Authors:** Francesca Abramo, Francesco Albanese, Silvia Gattuso, Alessandra Randone, Ivan Fileccia, Carla Dedola, Fabrizio Ibba, Paola Ottaiano, Emanuele Brianti

**Affiliations:** 1 Department of Veterinary Sciences, University of Pisa, 56124 Pisa, Italy; 2 Private Veterinary Laboratory “LaVallonea”, 20017 Passirana di Rho, Italy; francescoalbanese@laboratoriolavallonea.it; 3 Ambulatorio Veterinario Lionvet, 98168 Messina, Italy; silviagattuso@ymail.com; 4 Ambulatorio Veterinario, 10053 Bussoleno, Italy; alessandra.randone72@gmail.com; 5 Ambulatorio Veterinario Preneste, Centro Veterinario Specialistico, 00137 Rome, Italy; ivan.fileccia@gmail.com; 6 Centro Veterinario Ichnos, 09131 Cagliari, Italy; carladedola@hotmail.com; 7 Clinica Veterinaria Animal House, 09012 Capoterra, Italy; palmasoriano@me.com; 8 Clinica Veterinaria Firenze Sud, 50126 Florence, Italy; paolaottaiano@libero.it; 9 Department of Veterinary Sciences, University of Messina, 98168 Messina, Italy; ebrianti@unime.it

**Keywords:** *Leishmania*, cat, feline leishmaniosis, skin lesions, dermatology, systematic review

## Abstract

Feline leishmaniosis (FeL) is increasingly reported throughout the world and skin lesions predominate in the clinical picture. There are, however, few evidence-based data on cutaneous feline leishmaniosis and directions are strongly needed for a better management of the disease. In this study, we systematically reviewed what is currently known about the clinical dermatological presentation of FeL through analysis of the literature and, further, by adding unpublished cases managed by Italian veterinary dermatologists. Sixty-six feline cases of cutaneous leishmaniosis published in 33 articles between 1990 and 2020 met the inclusion criteria and were analyzed. Six unpublished cases of cutaneous FeL managed by Italian dermatologists were also reviewed. The majority of cases were reported from South America, followed by Europe and North America. Nodules were the most frequently reported clinical signs and the presence of Leishmania in lesioned skin was assessed mainly by cytology. A total of six Leishmania species have been identified as being responsible for skin lesions. Coinfections by FIV or FeLV were reported in 12.1% and 9.1% of the cases, respectively. Clinical data including treatment have been analyzed and discussed to provide directives for proper management of the disease for which cats may also serve as domestic reservoirs for human infections.

## 1. Introduction

Leishmaniosis is a disease caused by intracellular protozoa of the genus *Leishmania* (Kinetoplastida: Trypanosomatidae) and vectored by phlebotomine sand flies of the genus *Phlebotomus* and *Lutzomyia* in the Old and New World, respectively [1]. The disease in humans is included amongst the most important neglected diseases, and it has been the only tropical vector-borne disease endemic to southern Europe for decades [2]. In many epidemiological settings, dogs are regarded as the main source of *Leishmania* infection, but other domestic and wild animal species have also been implicated as reservoirs [3,4]. Due to the primary reservoir role and to the severe illness it may induce, leishmaniosis in dogs has been well studied and documented. Cats, on the other hand, have been regarded for many years as a less susceptible or even resistant animal species, and their role in the epidemiology of the disease is considered negligible.

The first report of experimentally induced feline leishmaniosis (FeL) in domestic cats dates back to 1984 [5], and despite the initial reluctance, cases of FeL have been increasingly reported throughout the world, especially in recent decades [6,7,8,9,10,11,12,13,14,15,16,17,18,19,20,21,22,23,24,25,26,27,28,29,30,31,32,33,34,35,36,37,38,39,40,41,42,43,44,45,46,47,48,49,50,51,52,53,54,55,56,57,58].

The rise in reports of FeL is likely connected with the development of feline medicine and the availability of more sensitive and specific diagnostic tests; nonetheless, adaptation to new hosts and changes in the feeding source of sand flies due to the widespread use of insect repellents in dogs as a way of prevention have been also suggested as drivers for the rise in FeL cases [59,60].

Recent epidemiological studies suggest that cats living in *Leishmania* endemic areas have similar exposition risk and prevalence data of dogs from the same area despite generally being asymptomatic or subclinical [61,62]. When associated with disease, FeL may include a variety of signs, with skin or mucocutaneous lesions and lymph node enlargement being the most common [63]. Indeed, dermatological lesions predominate in the clinical presentation of FeL and it has been estimated that they account for more than half of the clinical manifestations of FeL caused by *Leishmania infantum* (reviewed in Pennisi et al. **[63]**).

The epidemiological, serological and clinicopathological features of FeL have been thoroughly reviewed mainly by authors living in endemic areas [63,64,65,66,67,68]. More recently, a systematic review focused on evidence-based knowledge regarding the epidemiological aspects of FeL across the world [69], but, to date, no study has systematically reviewed, on a global scale, the dermatological presentations associated with FeL.

The awareness of veterinary practitioners of FeL has increased significantly, requiring more updated and robust evidence-based data to guide their diagnostic and therapeutic processes in managing a disease of great veterinary and medical concern.

In this study, we systematically reviewed what is currently known about the clinical dermatological presentation of FeL through analysis of the literature and, further, by adding unpublished cases managed by Italian veterinary dermatologists.

## 2. Results

### 2.1. Outcome of Case Selection

**Figure 1:**
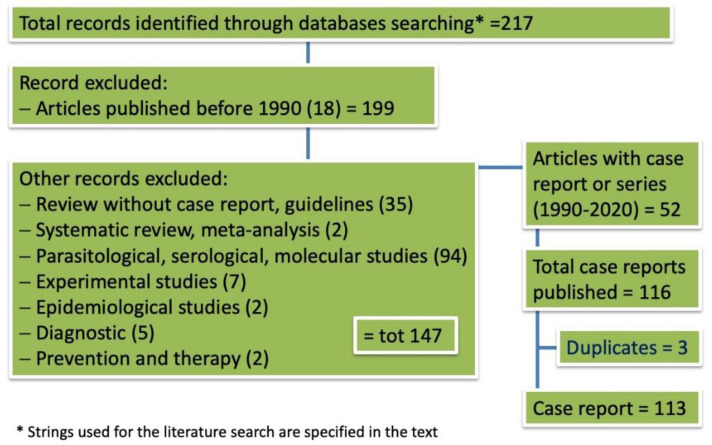
Flowchart illustrating the results of database search to find cases of feline cutaneous leishmaniosis.

**Figure 2:**
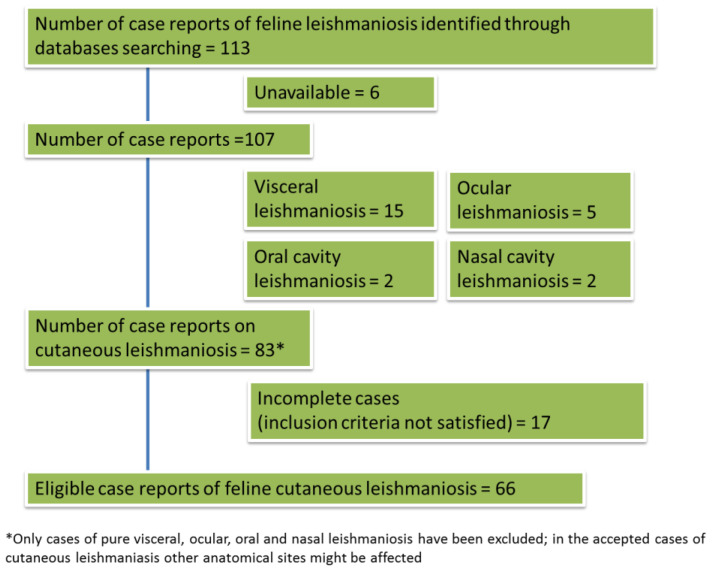
Flowchart illustrating the results of database search to find eligible cases of feline cutaneous leishmaniosis.

The PRISMA flowchart of the preliminary assessment from which we extrapolated the number of case reports or series is shown in Figure 1. Only articles published in the period between 1990 and 2020 were included in the systematic review due to the unavailability of the full-text version of the majority of papers published before 1990. The process of the selection of papers dealing with cutaneous FeL cases, leading to the final number of eligible manuscripts for the review, once non-cutaneous forms were excluded, is shown in Figure 2.

Two hundred and seventeen records were identified by using the selected strings, of which 165 were excluded because they were published before 1990 (18), as well as review papers including one systematic and one meta-analysis study (37), papers evaluating parasitological, molecular and serological data (94) and another 16 papers dealing with experimental infection, epidemiology, diagnostics, prevention and therapy of FeL (Figure 1).

Fifty-two papers, either case series or case reports, for a total number of 116 FeL cases, were published in the years between 1990 and 2020. Three of the above cases, however, were duplicated (cited twice in different papers by the same author), and for 6 cases, the full-text paper was not available, thus only 107 were reviewed.

From these 107 cases, 24 were excluded because they reported the diagnosis of visceral leishmaniosis without cutaneous involvement or exclusively localized in specific anatomical sites, namely the eye and the oral and the nasal cavities, and 83 cases of cutaneous leishmaniosis were extracted. After deeply reviewing each case and by critically evaluating if the diagnostic methods employed fulfilled the inclusion criteria (see Materials and Methods section below), another 17 cases were excluded. Thus, the systematic review was conducted on 66 eligible cases of FeL (Figure 2). Cases were from South America (37), Europe (23) and North America (6). European countries where cutaneous leishmaniosis has been detected were Spain (9), Portugal (5), Italy (4), France (3), Switzerland (1) and the UK (1). In South America, most of the cases have been described in Venezuela (22), followed by Brazil (13), Argentina (1) and French Guiana (Table 1).

### 2.2. Signalment and Clinical Presentation of Cutaneous Feline Leishmaniosis (66 Case Reports)

Fifty-six cases were domestic shorthair (DSH) cats, five were domestic longhair, two were Siamese, and for another three cats, the breed was not specified. The age of presentation was unknown for eight cases, nine were adults, while for the remaining 49 cases, the mean and median age were 5.8 and 5 years, respectively, with the youngest aged 6 months and the eldest 15 years. Gender information was available for 61 cases, and females were overrepresented with 39 cats, male with 22, yielding a ratio of 1.8. In half of the cases (50%, 33/66), the presence of coinfections was not investigated and, when available, data were: 23 cats (69.7%) were negative for FIV/FeLV; four (12.1%) [18,36,57] and three (9.1%) [17,22,35] tested positive for FIV and FIV/FeLV, respectively. One case was FIV positive and IgG positive for *Toxoplasma* [18], one case was FIP positive [19] and another cat tested positive for *Hepatozoon felis* and *Candidatus* Mycoplasma haemominutum [45].

**Figure 3:**
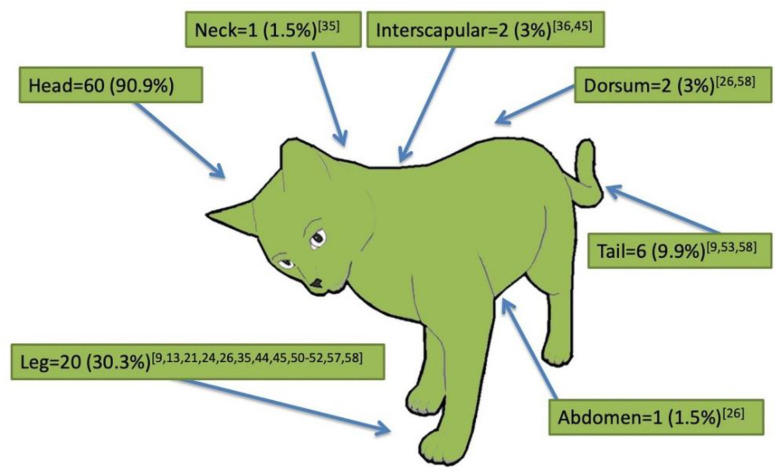
Anatomical distribution of lesions in the 66 reviewed cases of feline cutaneous leishmaniosis. References are reported as numbers between square brackets.

**Figure 4:**
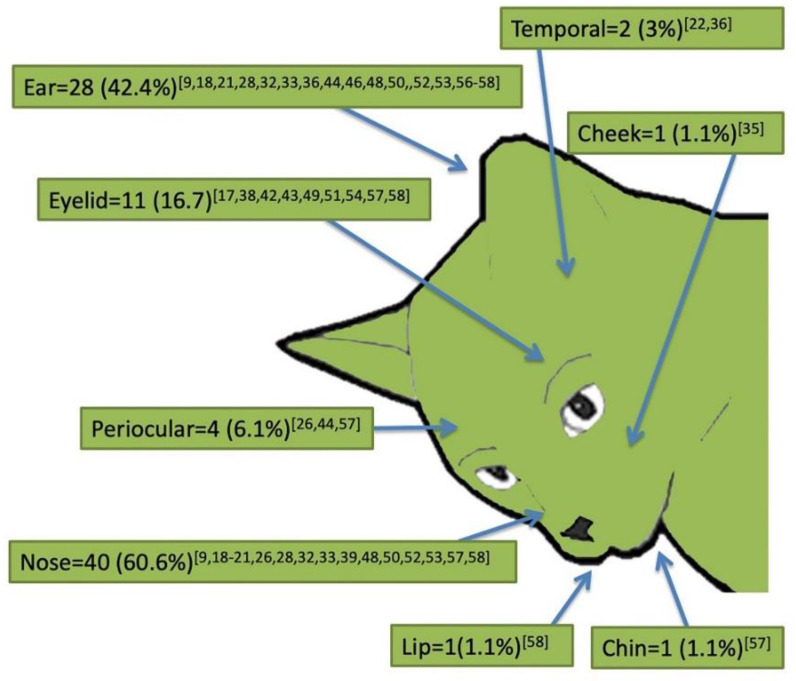
Anatomical distribution of lesions on the head in the 66 reviewed cases of feline cutaneous leishmaniosis. References are reported as numbers between square brackets.

The most often reported clinical signs were nodules (72.7%; 48/66), either intact or ulcerated (31.3%; 15/48), single (47.9%, 23/48) or multiple (52.1%, 25/48); ulcers or crusts (36.4%, 24/66), alopecia (9.1%, 6/66) and scales (7.6%, 5/66) were less frequently reported. The majority of animals (75.7%; 50/66) showed only one type of dermatological sign, while the others presented more dermatological signs such as alopecia, scales and nodules or ulcers. Dermatological signs were mainly reported on the head (90.9%; 60/66) and legs (30.3%; 20/66), followed by other anatomical regions (Figure 3). Within the head, lesions were more frequently described on the nose (60.6, 40/60), ears (42.4%, 28/60) and eyelids (16.7%, 11/60), and less commonly in other areas (Figure 4). Forty-one (62.1%) out of all described cases presented with only the cutaneous form without any visceral involvement, while in the other 25 (37.9%) cases, a visceral form including ocular (20%, 5/25) and nasal (8%, 2/25) involvement was reported. *Leishmania* was identified to species level in 44 (66.7%) cases. *L. infantum* was the sole species reported in cases from the Old World and in a few from the New World, whereas *L. mexicana* appeared as the species more frequently reported in the New World, with a total of 18 (40.9%) cases (Table 2). When ranked according to the *Leishmania* species, the visceral form was significantly higher when *L. infantum* was the causative species (χ^2^ = 9.0654; *P* = 0.0108; df = 2); conversely, no differences were observed in the frequency of dermatological signs other than nodules (e.g., ulcers, crusts, alopecia and scales) according to the *Leishmania* species (χ^2^ = 1.9245; *P* = 0.5882; df = 3).

Cytology on lesioned skin was the elective diagnostic method in most of the cases; indeed, it tested positive in 46 out of 49 cats (93.9%), and 35 of these presented with nodular lesions, while the other 11 showed ulcerative, alopecic and exfoliative dermatitis. In the cats in which cytology did not yield any visible amastigotes, the diagnosis was based on histology, immunohistochemistry (IHC) and/or PCR on samples collected from lesioned skin [50]. In the seventeen cases where cytology was not performed, the nodules were excised by surgery and amastigotes detected by histology (11) and/or by IHC and conventional or quantitative PCR (qPCR). Overall, histology was diagnostic for the presence of *Leishmania* amastigotes in 41 cases (62.1%), IHC was used and gave positive results in eight cases (12.1%), conventional PCR was positive in 31 (47%) feline skin lesions, while qPCR scored positive in another nine cases, but in only three cases was the number of copies made available in the paper [26,44,45].

As regards treatment, twenty-five (37.9%) cases underwent either surgery or medical therapy. Surgery alone was the chosen treatment in four cases, while it was used before medical therapy in one cat [24]. Medical therapy was initiated in 21 (31.8%) cases, nineteen (28.8%) cases did not receive any treatment, while in the remaining 22 (33.3%) cases, no data on treatments were available. Allopurinol was the most used drug; it was the only antiprotozoal drug in 12 cases and was used in combination with other drugs in the other six cases, as follows: in combination with meglumine antimoniate (four cases) [42,44,53,54]; followed by meglumine antimoniate (one case) [57]; followed by meglumine antimoniate and then miltefosine (one case) [49]. Meglumine antimoniate alone was used in three cases [53].

Coinfections were searched for in half of the cases (33) and found in 10, where FIV, FeLV, FIP, toxoplamosis, *Hepatozoon felis* and *Candidatus Mycoplasma haemominutum*, either alone or in various combinations, were diagnosed.

### 2.3. Unpublished Italian Cases

**Figure 5:**
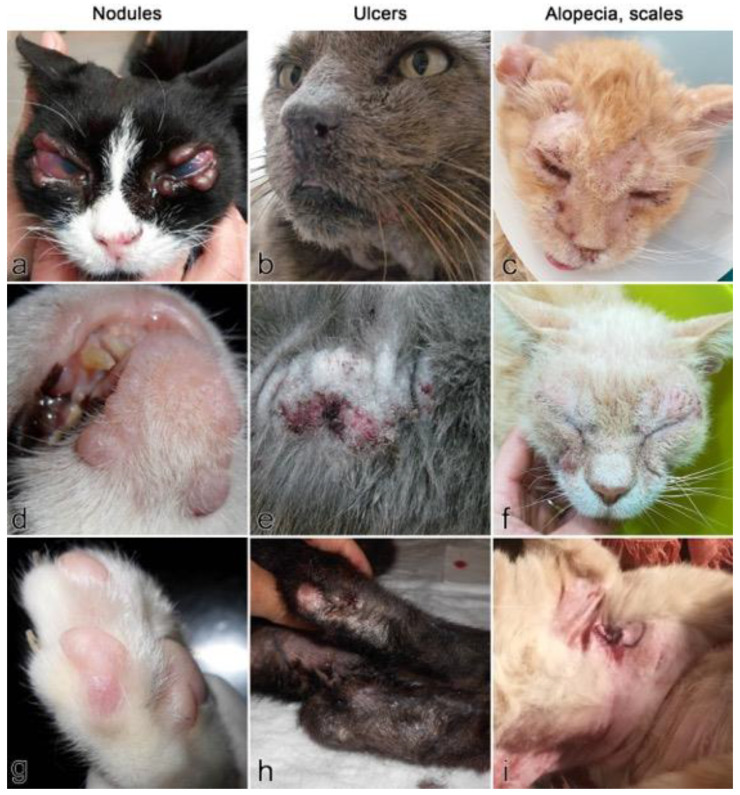
Clinical signs in the six unpublished cases of feline cutaneous leishmaniosis. Types of lesions reported are skin nodules (**a**,**d**,**g**), ulcers (**b**,**e**,**h**), alopecia and scales (**c**,**f**,**i**). (**a**) Case no. 3: multiple alopecic and non-ulcerated nodules on both eyelids. (**b**) Case no. 5: small ulcer on the left nostril. (**c**) Case no. 6: symmetrical alopecic, scaly and crusted dermatitis on the face. (**d**) Case no. 4: multiple alopecic, non-ulcerated nodules on the inferior lip and chin. (**e**) Case no. 5: wide alopecic and ulcerated lesion on the dorsum. (**f**) Case no. 1: symmetrical alopecic, scaly and crusted dermatitis on the face. (**g**) Case no. 4: small alopecic, non-ulcerated nodule adjacent to the lateral digit. (**h**) Case no. 2: bilateral alopecic, scaly and ulcerated lesions of the metatarsal region. (**i**) Case no. 1: diffuse abdominal alopecia, erythema and scaly dermatitis.

A total of six unpublished cases of FeL managed by Italian veterinarians and meeting the inclusion criteria were collected. Signalment and anamnestic data of the cases are shown in Table 3. At the time of diagnosis, the mean age was 9.6 years; two cats (no. 1, 6) were kept indoors, two had regular access to the outdoors (no. 2, 4) and two were kept outdoors permanently (no. 3, 5). All cats presented with both cutaneous and visceral signs; for one cat (no. 3), ocular involvement was also reported. Clinical presentation was different, being characterized by localized or diffuse lesions involving several body areas (Figure 5). As recorded in the published cases, the main affected region was the head (all cases), followed by ears (3/6), legs (3/6), dorsum (2/6) and ventral areas (1/6). The types of lesions consisted in nodules, ranging in diameter from a few millimeters to 1 cm in two cats (no. 3, 4); alopecia, scales, erosion and crusts were also described in the other four cats, where a clinical diagnosis of exfoliative dermatitis was made. Erosion and ulcers were also seen (no. 1, 5) and pruritus was reported in two cats (no. 1, 5), raising the clinical suspicion of an allergic disease. The diagnostic protocol included hair microscopy, skin scrapings and fungal culture for alopecic and scaly lesions and cytology for nodules. All cats were negative for fungi and parasites; for pruritic cats, flea prevention and food trials were carried out.

Cytology of the nodules scored positive for *Leishmania* amastigotes in five cats. Fine needle aspiration (FNA) of lymph nodes gave positive results for amastigotes in five cases, suggesting systemic involvement of the disease. Histology was decisive to achieve a diagnosis in two cases (no. 1, 2) and confirmed the presence of amastigotes in another three cases.

**Figure 6:**
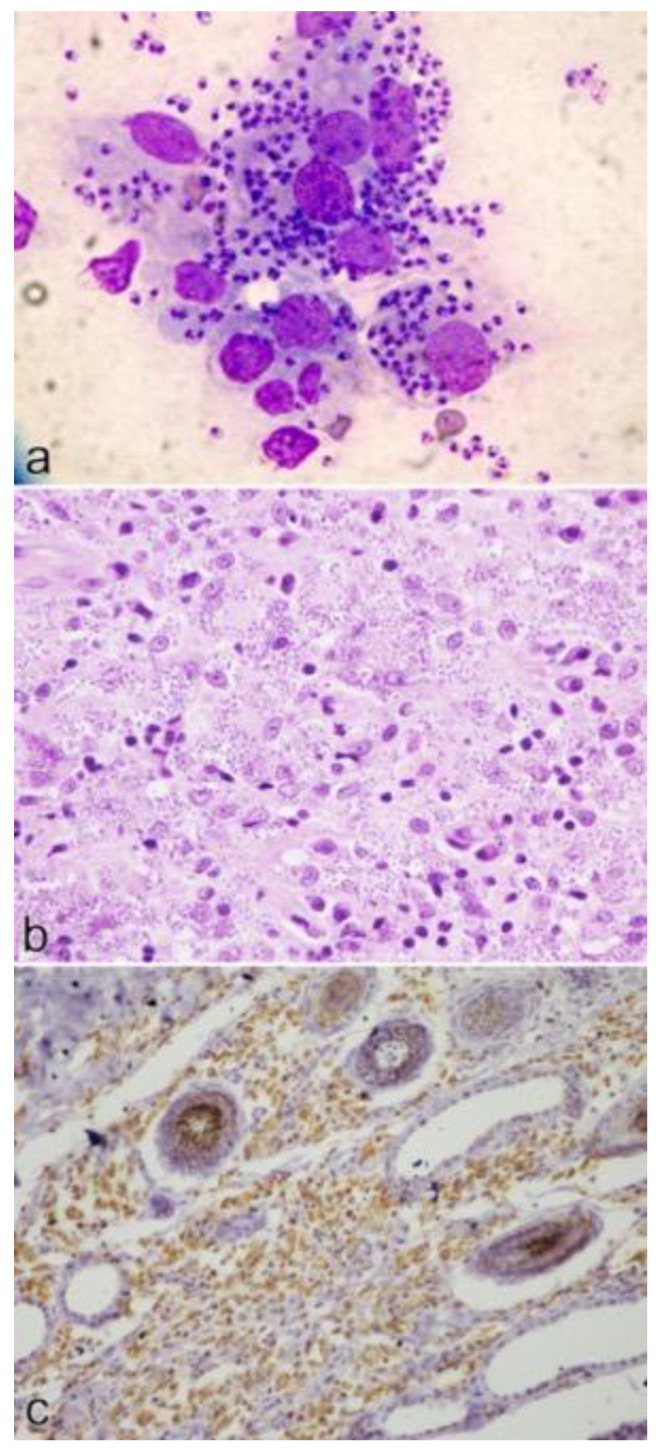
Cytological, histological and immunohistochemical findings in cases of feline cutaneous leishmaniosis: (**a**) cytology of a nodule from case no. 4: large macrophages containing numerous amastigotes; (**b**) histology of a nodule from case no. 4: nodular to diffuse granulomatous dermatitis, with lymphocytes and numerous macrophages filled with amastigotes; (**c**) immunohistochemistry of skin lesion from case no. 2: many protozoal organisms are positive by using a hyperimmune canine serum.

The main cytological, histological and immunohistochemical findings observed in the cases are shown in Figure 6. In case no. 2, IHC highlighted the presence of amastigotes within the tissue. *Leishmania* sp. was confirmed by IFAT in all but one case, with antibody titers ranging from 1:164 to 1:3200. In case no. 2, *L. infantum* was identified and leishmanial DNA quantified as 104 copies/PCR-2500.

All cases underwent an antiprotozoal therapy regimen with allopurinol alone (3/6), allopurinol associated with antimonial salts (2/6) or with domperidone (1/6). Other drugs were used to treat pruritus or concomitant disease (e.g., ocular disease). Complete remission of the dermatological signs was seen in three cats in a variable period of time ranging from 1 month to 1 year (Table 3).

## 3. Discussion

This review collected and analyzed a total of 66 FeL cases published between 1990 and 2020 in which *Leishmania* spp. was the cause of skin lesions, thus providing a large amount of evidence-based data for a better understanding of the etiology, clinical presentation, diagnosis and treatment of a disease increasingly reported in cats living in endemic areas.

The growing interest in FeL and the rise in cases is shown by the mean number of published articles per year (13, range 6–20) in the last decade of analysis, which is larger than that recorded in the previous decade (3, range 0–6). Interesting, although many of the published articles were from South America, with Brazil accounting for more than a third of all reported cases, Europe follows in second place, supporting the importance and the spread that this disease has achieved in the continent [69].

From the initial 199 case reports found in the literature, we extracted and submitted to systematic review a smaller number, mostly due to incomplete or poor descriptions of clinical signs and/or failing to meet diagnostic criteria for cutaneous leishmaniosis. In fact, we set as compulsory the detection of the parasite in the lesioned skin, in order to consider it the causative agent of the lesions. Consequently, we excluded those cases with dermatological signs in which the diagnosis was based on the serology alone or detection of the parasite in internal organs such as lymph nodes; in some of these cases, the lesions were cured after protozoal therapy, but a causative effect could not be proven. As evidenced by the cases analyzed in this study, the detection of the parasite in the skin can be easily achieved by: (i) cytology, either using FNA from nodular lesions, direct imprint on ulcerative lesions, imprint of the reverse edge of crusts once they are removed or by FNA biopsy of the sub-epidermal dermis using an insulin needle; (ii) histology or (iii) IHC on skin biopsies, if amastigotes are few in number and difficult to detect in routine Haematoxylin & Eosinstained sections.

Immunohistochemistry is performed by using specific antibodies [70,71,72]; however, Tafuri et al. [73] showed an alternative useful method that allows for the detection of small numbers of amastigotes. The method relies on the use of a streptavidin–biotin method with canine hyperimmune serum as the primary antibody. Cross-reactivity between human or canine anti-*Leishmania* hyperimmune serum and fungal forms with IHC has been reported and might occur in dogs, however, a careful observation of organism morphology or the use of special stains (Periodic acid-Schiff or Grocott) may help to distinguish the different infectious agents. The same might occur in cats and the method has already been successfully applied for the recognition of tissue amastigotes in feline patients [40]. Tafuri’s method was successfully performed in one of the six unpublished FeL cases, and the method was applied to better characterize the histopathological findings and further supports the validity of this method with cat tissues.

PCR is also a method that can be used to prove the presence of kinetoplast DNA in tissues. A quantitative molecular method is necessary to overcome the problem that skin might occasionally be positive with conventional PCR in infected animals, though a cut-off of the parasite load for determining it as the causative agent of lesions has not been determined yet. Quantification of the parasite load has been proposed quite recently to screen cat conjunctival swabs [74] and was rarely used for diagnostic purposes in the case reports analyzed here. In particular, the number of parasites has been indicated by Dalmau et al. [26], Basso et al. [44] and Attipa et al. [45], with values expressed as per tissue analyzed [26,44] or by relative copy numbers [45]. In another two papers, the authors only mentioned positivity in qPCR from skin tissue but did not provide the loads [50,57]. Additionally, real-time PCR can be used not only for diagnostic purposes but also with the intent to follow-up cats under treatment [45].

**Figure 7:**
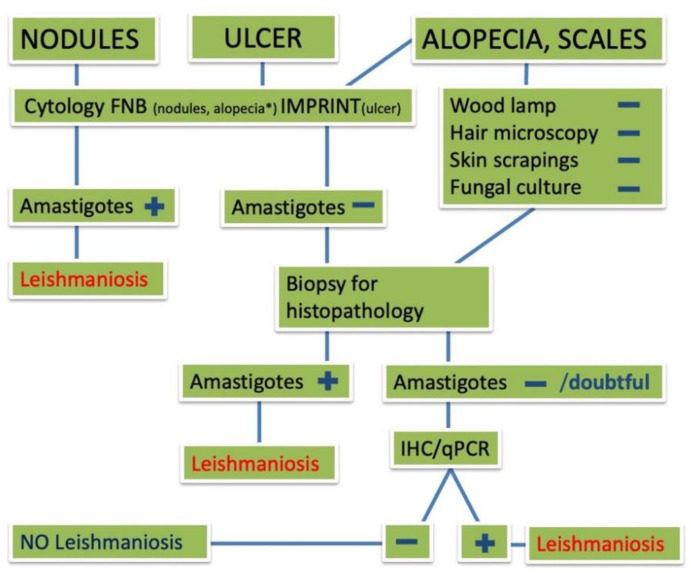
Sequence step algorithm for the diagnosis of feline cutaneous leishmaniosis according to the type of skin lesion. FNB = fine needle biopsy; IHC = immunohistochemistry; qPCR = quantitative polymerase chain reaction.

In all cases included in this systematic review, FNA was the most used method of diagnosis due to the large number of cats presenting with nodules. This may also be the reason for the high prevalence of nodular forms of FeL in the literature, as the other clinical signs were far more difficult to diagnose without histopathology and/or IHC and/or real-time PCR. To assist clinicians in the diagnosis of feline cutaneous leishmaniosis, an algorithm suggesting the diagnostic protocol according to the type of skin lesion (e.g., nodule, alopecia or ulcer) is proposed in Figure 7; once amastigotes have been detected by cytology or histology/immunohistochemistry, further species identification (i.e., PCR) can be applied, if necessary.

Although the list of differential diagnoses is quite large for the nodular forms, including both infectious diseases, such as fungal, bacterial or protozoal diseases, and non-infectious diseases, such as sterile granulomas, metabolic accumulations (i.e., xanthomatosis) and neoplastic disease, FNA is an easy and rapid diagnostic technique for cytological diagnosis [75]. On the other hand, alopecic, exfoliative, erosive and ulcerative clinical presentations all need a more complex approach that most often lead to a biopsy for histopathology, IHC or qPCR. The most often cited differential diagnoses for these presentations are dermatophytosis, demodicosis, hypersensitivity dermatitis, thymoma or non-thymoma-associated exfoliative dermatitis, idiopathic lymphocytic or mucinotic mural folliculitis, epitheliotropic lymphoma/cutaneous lymphocytosis and exfoliative dermatitis associated with FIV.

From the data presented here, it seems that there is no breed predilection for developing cutaneous leishmaniosis, and this might reflect the vast majority of non-purebred cats all over the world. As in dogs, adult cats were mainly affected compared to kittens, that seem less exposed.

As mentioned above, nodules were the most frequent skin lesions; although these were reported in several body areas, they were more frequent on the head, both as unique and multiple lesions, especially on the nose, eyelids and pinnas. In the case in which the unique lesion is a nasal ulcer, squamous cell carcinoma is the main differential. Only seven cases with alopecic and exfoliative dermatitis due to *Leishmania* were found to fulfill inclusion criteria for the diagnosis, and four out of the six unpublished cases herein reported presented with alopecic, exfoliative and crusty dermatitis, thus implementing data on this type of clinical presentation. As already mentioned above, this presentation is observed in many other diseases whose diagnosis often needs a skin biopsy or other diagnostic tool (i.e., thoracic ultrasonography, X-ray or computed tomography for thymoma-associated exfoliative dermatitis); however, in all the cases described, the parasitic load was high and performing cytology by FNA may also be of great value in cases of alopecic and exfoliative dermatitis associated with FeL.

A total of six *Leishmania* species have been identified as being responsible for skin lesions in the feline cases analyzed here. The species have been described according to their geographical endemicity, with the largest variety in South America, while *L. infantum* seems to be the sole species responsible for disease in European countries. It is noteworthy that all six species are of zoonotic concern, either as an agent of localized cutaneous leishmaniosis (*L. amazoniensis*, *L. brasiliensis*, *L. infantum*, *L. mexicana*, *L. panamensis*, *L. venezuelensis*), diffuse cutaneous leishmaniosis (*L. amazoniensis*) or visceral leishmaniosis (*L. infantum*), further supporting the potential reservoir role of domestic cats in the epidemiology of human and animal leishmaniosis. Besides cutaneous forms, visceralization has been reported in many of the cases caused by *L. infantum*, conversely, it occurred rarely in those caused by *L. brasiliensis* (1/6) or *L. mexicana* (1/18), paralleling the cutaneous species trophism already described in other animal species, including humans [76].

Allopurinol was used in half of the reviewed cases that underwent medical therapy and was the drug of choice for all the Italian cases described here. According to the latest recommendations on FeL, allopurinol is the sole drug with extensive clinical data available to be considered effective [67]. The drug is usually well tolerated, however, a strict monitoring of the health status of cats under this treatment, including urinalysis and kidney ultrasound, is strongly recommended [67]. Antimonial salts were also frequently used in association with allopurinol, and, in a few cases, used alone with an apparently higher frequency of relapses (Paniz 2019) [53]. Lastly, domperidone has been used in association with allopurinol in one case previously reported by Maia et al. (2015) [39] and in one of the Italian cases, thus likely contributing to the positive clinical outcome. Domperidone, a dopamine D2 receptor antagonist, is known to promote cell-mediated immunity in dogs in which it reduces the risk for seronegative patients to develop an active infection and improves clinical signs and disease progression in already affected patients [77,78]. Although no studies have assessed the efficacy of domperidone against FeL, its use in feline patients seems promising. Finally, despite not being commonly adopted, insect repellents should be used in antiprotozoal therapy in order to combat the reservoir role of diseased cats and to prevent re-infections in exposed animals [79].

## 4. Materials and Methods

### 4.1. Searching Strategy

We review the available information published to date for any case reports or series of FeL according to the Preferred Reporting Items for Systematic Review and Meta-Analysis (PRISMA) [80] by searching through the following databases: PubMed Web, CAB Abstracts and Google Scholar, without any language restriction. The strings used for the search were: “*cat” or “cats” or “feline”* and “*cutaneous”* or *“skin”* and *“visceral”* and “*leishmaniosis”* or *“leishmaniasis”* or *“Leishmania*” for PubMed Web and CAB Abstracts and “*feline cutaneous leishmaniosis”* and “*feline cutaneous leishmaniasis*” for Google Scholar. Each of the selected papers was reviewed by one of the authors (F. Abramo) for any possible additional publications that could not be captured by the aforementioned search strategies. The search started in October 2020 and ended on 1 December of the same year.

When detailed clinical case information was not reported, the publication was excluded due to the inadequate quality of the material for the review and unlikelihood to guarantee that the cases met inclusion criteria (see below). Papers were excluded from the study for the following reasons: abstracts for which the full text was unavailable, congress proceedings, experimental studies, books and duplicates. Manuscripts describing non-cutaneous FeL, namely, visceral, ocular, nasal and buccal involvement, were also excluded.

Case reports or series of cutaneous FeL were included if they fulfilled the following inclusion criteria: (i) *Leishmania* amastigotes were directly detected from skin lesions by smears or histopathology and (ii) amastigotes were detected from the skin by IHC and/or *Leishmania* DNA was detected from the lesioned skin by PCR methods.

Additionally, unpublished dermatological cases of FeL recently managed by the authors and fulfilling the inclusion criteria were also included in this review and analyzed.

### 4.2. Statistical Analysis

The typology of data collected (e.g., case report or case series) impaired the meta-analysis approach. Descriptive statistics were carried out for the data collected and, when permitted, associations between variables and outcomes were assessed using frequency analysis. All data analyses were performed using the software JASP v. 0.14.1 (JASP Team 2020, https://jasp-stats.org).

## 5. Conclusions

Feline leishmaniosis is a disease increasingly reported throughout the world. Domestic cats are exposed and may get infected with a variety of *Leishmania* species according to the geographical endemicity, thus contributing to the epidemiology of the disease as reservoirs. Cutaneous lesions are the sole or the most recurrent clinical signs in FeL, with nodular lesions being the most frequently reported for all cases and *Leishmania* species. Determination of the presence of parasites in the lesioned skin through cytology, histology or molecular tools is compulsory for attributing the etiology and for discriminating among other pathological conditions. Coinfections (e.g., FIV and FeLV) should be always investigated, as they may worsen the clinical picture and impair prognosis.

Data on the treatment of FeL are still limited since only a minority of cases reviewed here underwent therapy; however, allopurinol, either alone or in combination with other antiprotozoal drugs, represented the main choice in the majority of cases and yielded curative effects in many of them. Despite not being commonly adopted, the use of insect repellents as a measure of prevention against infection and/or for the control of the reservoir role should be encouraged in cats living in or traveling to *Leishmania* endemic areas.

## Figures and Tables

**Table 1:** Cases of feline cutaneous leishmaniosis published between 1990 and 2020 according to country, type of study and *Leishmania* species.

Continent	Country	Type of Study (No.)	No. of Cases	Breed (No.)	Species (No.)	References
Europe	France	Case report (3)	3	DSH (3)	*L. infantum* (3)	[14,22,36]
	Italy	Case report (4)	4	DSH (4)	*L. infantum* (2); *Leishmania* sp. (2)	[17,18,35,51]
	Portugal	Case report (5)	5	DLH (1); DSH (4)	*L. infantum* (3); *Leishmania* sp. (2)	[39,42,44,49,54]
	Spain	Case report (2); Case series (1)	9	DSH (7); Siamese (2)	*L. infantum* (1); *Leishmania* sp. (8)	[26,57]
	Switzerland	Case report (1)	1	DSH (1)	*L. infantum* (1)	[38]
	United Kingdom	Case report (1)	1	DLH (1)	*L. infantum* (1)	[45]
North America	USA	Case report (1); Case series (1)	6	DLH (1); DSH (5)	*L. mexicana* (6)	[32,46]
South America	Argentina	Case report (1)	1	DLH (1)	*L. braziliensis* (1)	[43]
	Brazil	Case series (4); Case report (5)	13	DLH (13)	*L. amazonensis* (3); *L. braziliensis* (2); *L. infantum* (3); *L. panamensis* (1); *Leishmania* sp. (4)	[13,19,20,21,48,52,56,58]
	French Guiana	Case report (1)	1	DLH (1)	*L. braziliensis* (1)	[33]
	Venezuela	Case series (3)	22	DSH (19); ND (3)	*L. braziliensis* (2); *L. mexicana* (12); *L. venezuelensis* (2); *Leishmania* sp. (6)	[9,50,53]

Legend: DSH = domestic short hair; DLH = domestic long hair.

**Table 2:** Forms and dermatological signs in cases of feline cutaneous leishmaniosis according to *Leishmania* species.

*Lesihmania* Species	Cases	Forms		Sign				References
		Cutaneous	Visceral	Nodule (Ulcerated)	Ulcer/Crust	Alopecia	Scale	
*L. amazonensis*	3	3	-	3 (0)	1	-	-	[21,28,56]
*L. brasiliensis*	6	6	1	6 (4)	1	-	-	[20,33,43,50]
*L. infantum*	14	14	11	11 (4)	4	2	-	[14,17,19,22,24,36,38,39,44,45,51,54,58]
*L. mexicana*	18	18	1	11 (4)	7	-	1	[32,46,50,53]
*L. panamensis*	1	1	-	1 (0)	-	-	-	[13]
*L. venezuelensis*	2	2	-	2 (0)	-	-	-	[9]
*Leishmania* spp.	22	22	14	14 (3)	11	4	4	[9,18,26,35,42,48,49,50,52,53,57]

**Table 3:** Clinical presentation, diagnosis, comorbidity and therapy of the six unpublished cases of feline cutaneous leishmaniosis managed by Italian dermatologists.

Case No.	Breed, Gender, Age (yrs)	Region	C–V	Localization	Type of Lesion	Citology		Histology	IFAT Titer	PCR	Comorbidity	Therapy	Treatment Outcome
						Skin	L.N.						
1	DSH, M, 10	Sicily	C–V	Head (periocular), dorsum, groin, axillae, abdomen	Alopecia, erosion, pruritus	−	+	+	1:160	PCR +ve	FIV −ve, FeLV −ve	Allopurinol (10mg/kg BID 6 months) + Cyproheptadine 2 mg	Alive, under therapy for pruritus, remission and retreated with allopurinol
2	DSH, M, 9	Sardinia	C–V	Head, ear, neck, leg (metatarsus hock)	Alopecia, exfoliation	−	+	+	−	qPCR +ve	FIV +ve, FeLV −ve	Allopurinol (10mg/kg BID) + Domperidone (0.5 mg SID)	Alive, second cycle therapy after 6 months, recovered 12 months after the first presentation
3	DSH, F, 12	Piemonte	C–V	Head (eyelid, chip, nose, nares)	Multiple ulcerated nodules	+	+	+	1:1280	PCR +ve	FIV +ve, FeLV −ve	Allopurinol (10mg/kg BID) + Acetate prendnisolone (2 mg SID)	Complete remission in three months, died one year after from chronic renal disease
4	DSH, F, 4	Lazio	C	Head (eyelid, lip, chin), leg (digit)	Multiple nodules	+	−	+	1:320	PCR +ve	FIV −ve, FeLV +ve	Allopurinol (5 mg BID)	Complete remission in three weeks, no replapse, died after one month from lymphoma
5	DSH, M, 12	Sardinia	C–V	Head (nose, nares), ear, leg, dorsum	Alopecia, ulcer, crust, pruritus	+	+	−	1:1280	PCR +ve	FIV +ve, FeLV −ve	Allopurinol (10mg BID) + Antimonial salts (50 mg SID)	Complete remission in one month, died soon after from car crash
6	DSH, M, 11	Tuscany	C–V	Head (periocular) ear	Alopecia, crust, exfoliation	+	+	+	1:3200	PCR +ve	FIV +ve	Allopurinol (10mg BID) + Antimonial salts (15 mg SID 30 days)	Complete remission after 6 months, euthanasia for hypertrophic myocardiopathy and thrombosis

Legend: IFAT = immunofluiorescence antibody test; L.N. = lymph node; DSH = domestic short hair; F = female; M = male; C = cutaneous form; V = visceral form; L.N. = lymph node; +ve = positive; −ve = negative; BID = twice a day; SID = once a day.

## Data Availability

All the datasets supporting the findings of this study are available from the corresponding author (F. Abramo) on request.
